# Artificial Intelligence as a Scientific Copilot in Analytical Chemistry: Transforming How We Write, Review, and Publish

**DOI:** 10.1021/acs.analchem.5c03767

**Published:** 2025-09-15

**Authors:** Adrián Fuente-Ballesteros, Victoria Samanidou, Seyed Mosayeb Daryanavard, Ana M. Ares, José Bernal

**Affiliations:** 1 Analytical Chemistry Group (TESEA), I. U. CINQUIMA, Faculty of Sciences, University of Valladolid, Valladolid 47011, Spain; 2 Laboratory of Analytical Chemistry, School of Chemistry, 193601 Aristotle University of Thessaloniki, Thessaloniki GR 54124, Greece; 3 Deparment of Chemistry, Faculty of Science, 185136 University of Hormozgan, Bandar-Abbas 79511, Iran

## Abstract

Artificial intelligence (AI) is increasingly present across all phases of analytical chemistry, not only in experimental workflows but also in the way scientific writing is produced, evaluated, and published. This perspective offers a critical reflection on the growing use of AI tools as writing copilots in the field, focusing on novel yet underexplored practices such as literature review support, manuscript drafting, and AI-assisted peer review. While tools like ChatGPT, SciSpace, and Grammarly are becoming commonplace in manuscript preparation, their integration also raises important concerns about authorship transparency, originality, and the homogenization of scientific voice. The article highlights both the opportunities and limitations of these technologies. A comparative analysis is presented to summarize the main strengths, weaknesses, opportunities, and threats associated with AI use in scientific communication. This work advocates for the responsible adoption of these tools, the development of ethical guidelines, and the inclusion of AI training in analytical chemistry curricula. By encouraging the scientific community to reflect on these changes collectively, we expect to ensure that AI enhances, rather than undermines, the critical thinking and creativity that define scientific authorship.

## Graphical abstract

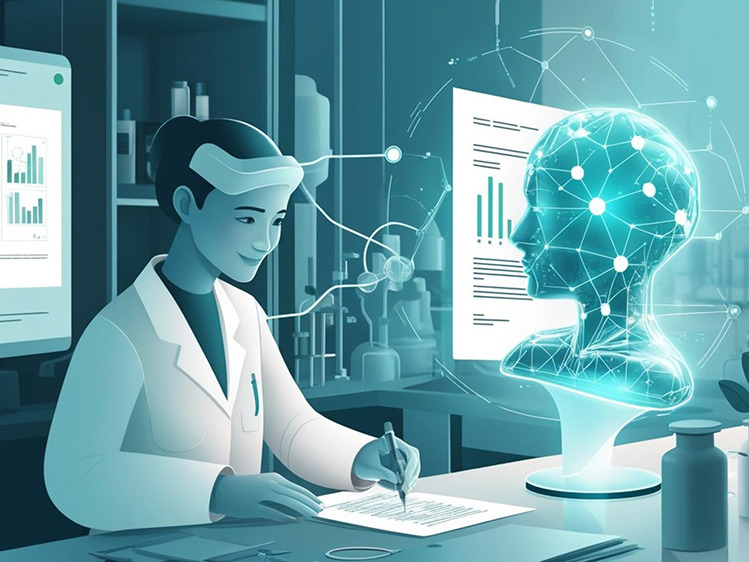

## Growing Presence of AI in Analytical Chemistry

Artificial intelligence (AI) is no longer a futuristic promise; it is a present-day reality in analytical chemistry. Once limited to niche applications in data analysis and statistical modeling, AI is now being routinely employed in method development, sample classification, and instrumental optimization. Its capacity to handle complex data sets, identify patterns, and accelerate decision-making has made it an essential ally in modern analytical workflows.^1^ Yet, its influence extends well beyond the laboratory bench. One of the most striking and underexplored developments is the growing presence of AI in scientific communication, particularly in writing and publication processes. Tools based on natural language processing are now being used to assist in the drafting of scientific manuscripts, generate summaries of research articles, respond to reviewer comments, and even improve the clarity and style of academic texts. In analytical chemistry, these tools are starting to quietly change the way we write and share science, something that has traditionally followed well-established rules. In just a few years, AI has gone from being a novelty to becoming part of almost every stage of the research process. The following sections examine the current applications of AI in the field, with attention paid to its integration in scientific writing, and offer a critical reflection on the strengths, challenges, and ethical considerations arising from this ongoing evolution. To the best of our knowledge, this is the first work to explore the role of AI as a scientific copilot in the context of analytical chemistry.

## Fields and Applications of AI in Analytical Chemistry Research

AI is gradually changing the way we work and think in analytical chemistry, particularly in the way complex data is processed and interpreted. Traditional approaches, which often rely on expert-driven and time-intensive interpretation, are increasingly being complemented (or replaced) by advanced algorithms capable of extracting relevant information in a faster, more reliable, and often more insightful manner. AI-based tools not only accelerate workflows but also enhance reproducibility, especially when dealing with heterogeneous and high-dimensional data sets. AI has been applied for optimization and data interpretation of several analytical approaches, including biosensors, microfluidics, chromatography, mass spectrometry, Raman, hyperspectral imaging, and near-infrared spectroscopy.^2^ Here, we provide a few examples of AI applied to different analytical applications:

i Spectral data processing and statistical analysis: Machine learning algorithms and neural networks are routinely used to deconvolute complex spectral signals, allowing for faster and more accurate compound identification.^3,4^

ii Predictive modeling: AI improves the construction of robust calibration models for quantitative analysis and prediction, particularly in food, environmental, and clinical matrices.^5–8^

iii Experimental design and optimization to optimize instrumental conditions and analytical protocols, often reducing the number of experimental runs required.^9–11^

iv Sample classification and pattern recognition: Supervised and unsupervised learning methods are used for clustering, classification, and feature selection in complex matrices, improving the discrimination of analytes or sample origins.^12^
v Real-time quality control and anomaly detection: AI models trained on production or experimental data streams can identify deviations from normal conditions, making them valuable in biomedical applications.^1^
vi Automation of instrumentation and laboratory operations: From robotic sample handling to automated data processing, AI is helping in the development of autonomous laboratories and smart instrumentation systems.^13,14^

vii Sensor development and smart detection systems: AI-enhanced chemical sensors are being developed for environmental monitoring, point-of-care diagnostics, and real-time detection of trace analytes.^15^
viii Green analytical chemistry: AI supports the selection of environmentally friendly solvents and processes through predictive sustainability scoring systems and the design of miniaturized or waste-minimizing methods.^16,17^

ix Spectroscopic profiling in food analysis: AI-assisted statistical models accelerate authentication and adulteration detection.^18,19^

x Metabolomics and omics-based profiling: Advanced AI algorithms are increasingly applied to metabolite identification and profiling improving accuracy and interpretation of biological samples.^20^

Most applications to date focus on data-intensive or technically complex tasks, where AI’s ability to manage large volumes of information offers clear benefits. Yet, one area where AI is now starting to make a noticeable impact is scientific communication, an aspect that remains less discussed but is increasingly relevant to the future of the field.

## AI as a Scientific Copilot in Analytical Chemistry

While AI has firmly established its role in the experimental and computational aspects of analytical chemistry, a more subtle yet equally transformative shift is taking place in the context of scientific writing. Beyond data analysis and method optimization, AI is increasingly serving as a copilot for researchers during the composition, refinement, and organization of scientific manuscripts. This evolution signals a new phase in how analytical knowledge is constructed and disseminated. The recent study by Kalyniukova et al. provides a timely example of this trend.^10^ In this work, the authors investigated the capabilities of ChatGPT 3.5 in supporting the development of an analytical procedure for the extraction and determination of bioactive compounds in plant samples. The researchers aimed to involve AI in as many stages of the process as possible, from experimental planning and selection of optimal conditions, to data interpretation and assessment of the method’s environmental sustainability. They also examined whether, based on experimental data, AI could independently produce a high-quality scientific manuscript, potentially performing tasks traditionally carried out by humans. They concluded that AI can be a good tool for an analyst, as it can relieve him of tedious and routine tasks, but it cannot replace the analyst.

In parallel, AI tools have also gained traction in other writing-related tasks. Graphic design platforms such as Canva and Microsoft Designer now incorporate AI to generate graphical abstracts, visual summaries, or table of contents figures, often enhancing the visual communication of results. Similarly, platforms powered by AI are used to detect similarities and reduce the risk of unintentional plagiarism, providing a layer of integrity to the publication process.^21^

However, this section focuses on some areas that, despite their growing relevance, have received comparatively less attention in recent discussions of the use of AI in analytical chemistry. These tools, often working quietly in the background, are starting to change the way researchers in analytical chemistry approach and experience the writing process.

### Literature Review Support

One of the most time-consuming and cognitively demanding stages in scientific writing is the review of the existing literature. Traditionally, constructing a comprehensive and balanced state-of-the-art section requires not only access to multiple databases but also a high level of domain expertise to assess the relevance and novelty of the sources. In recent years, AI-based tools have begun to serve as powerful copilots in this process, enhancing researchers’ ability to retrieve, organize, and synthesize scientific information efficiently. Platforms such as Elicit, Research Rabbit, and Scite exemplify this trend. These tools use natural language processing and machine learning algorithms to automatically identify potentially relevant publications based on input queries, keywords, or even entire abstracts. Unlike traditional search engines, AI-driven systems can suggest articles that are conceptually related, even when they do not share exact terminology, allowing for broader and sometimes more creative explorations of the literature.

Beyond simply locating relevant papers, AI also supports the classification and organization of articles, assisting researchers in mapping out the analytical landscape of a given topic. These systems can extract and summarize methodologies, analytical parameters, and key results, often presenting them in structured formats, such as comparison tables or visual summaries. Additionally, some tools offer metadata extraction, enabling the identification of trends, cocitation networks, or research clusters, insights that would require substantial time and expertise to compile manually. Nevertheless, the use of AI in literature reviews is not without risks. By relying on popularity metrics, citation counts, or publication recency, these tools may unintentionally bias the review toward well-known or recent studies, potentially overlooking foundational but less cited work or innovative contributions published in niche journals. This risk highlights the importance of critical human oversight in the curation of sources and the interpretation of algorithmically suggested content.

Given our expertise, AI can significantly streamline the review process. However, its contribution should be viewed as complementary rather than substitutive, supporting researchers without replacing the analytical judgment that defines robust scientific scholarship.

### Drafting and Writing Scientific Articles

The drafting of scientific manuscripts is a critical phase in the communication of research findings, often requiring not only domain expertise but also a high level of linguistic precision and structural coherence. In recent years, large language models (LLMs) such as ChatGPT and Gemini, along with AI-powered tools such as NotebookLM and Perplexity, have served as writing copilots for researchers in analytical chemistry, assisting in the generation of text and the organization of manuscripts with speed and fluency. These tools are commonly employed to produce initial drafts of key sections based on minimal prompts or preliminary input data.^10^ The output responses are generally accurate but often lack depth. It is well-known that the quality of AI-generated output strongly depends on three key factors: (a) the quality and specificity of the prompt, (b) the training of the AI-Chatbot, and (c) whether the user is operating with a premium or free version of the tool.^22^ Some specific examples of how AI can help to draft an article include: (i) selecting relevant keywords, (ii) proposing a table of contents, (iii) generating comparative tables, (iv) suggesting linking words, verbs, or phrases, (v) checking structural completeness, (vi) organizing abbreviations, (vii) arranging references according to the target journal’s author guidelines, (viii) homogenizing and analyzing large amounts of data, (ix) recommending suitable journals based on the content and scope of the work, (x) proposing alternative titles and subtitles for greater clarity, and (xi) suggesting paragraph reorganization to improve logical flow.

Despite the acceptable quality of these AI-generated responses, in agreement with the opinion of Kalyniukova et al.,^10^ they are not sufficient for the resulting text to be used verbatim as a section of a scientific article. Another increasingly common use is the translation and reformulation of scientific text into English, particularly for non-native speakers.^23^ AI-powered tools are capable of improving grammar, vocabulary, and academic tone, making scientific communication more accessible and efficient for the broader research community.

A growing concern, however, is the generation of fabricated references from language models. When prompted to add citations, AI may produce entries that appear legitimate, complete with titles, author names, journal references, and even DOIs. At first glance, these references may seem credible, but upon verification, they are often found to be entirely fictitious or unrelated to any real publication. This phenomenon poses a serious threat to scientific integrity and highlights the importance of manually verifying all AI-generated citations before inclusion in a manuscript.

Despite these advantages, the growing reliance on AI in manuscript drafting introduces potential drawbacks. One of the most significant concerns is the homogenization of scientific styles and the repetitive use of certain keywords. Texts generated by AI, while often grammatically correct and structurally coherent, may lack the depth, originality, and critical voice typically found in expert scientific writing. Some structures and expressions are often, though not exclusively, associated with AI-generated content (see Table 1). While the presence of such phrases or their derivatives does not necessarily indicate that a manuscript was written by AI, their repetitive and uncritical use may be indicative of mechanical writing, lacking reflection, or a personal voice. Sometimes this can lead to generic and repetitive formulations that dilute the originality and interpretive depth of the manuscript. Even the presence of AI can be easily detected by the excessive use of certain punctuation marks, such as the em dash (—). The em dashed line can function like a comma, a colon, or parentheses. Sometimes, the writer does not want the interruption of a pair of parentheses or the deliberate pause of a colon, so they use an em dash instead. When AI is used continuously to generate paragraphs, a bunch of repetitive em dashes (—) thrown in can make the reader frown and suspect AI-generated content. However, it can be difficult to determine whether a piece of text was written by an AI or a human, as AI tools are designed to generate human-like text.^21^ Therefore, while AI can accelerate the drafting process and overcome language barriers,^23^ its output requires careful revision to ensure that the scientific message remains accurate and truly reflects the researcher’s voice.

**Table 1:** Common Detected AI-Generated Lexical Items by Linguistic Category in Scientific Texts

category	examples
verbs	play a crucial role; contribute positively; entail; evolve; navigate; articulate; leverage; encompass; facilitate; grasp; delve into/deeper; showcase; underscore; embark; excel; cultivate; elevate; resonate; provide valuable insights; endeavor; cater; encounter; embrace; harness; reshape; necessitate; garner; resemble; embed; gather; emerge; deem to be
adverbs	precisely; inherently; consistently; succinctly; seamlessly; profoundly; undeniably; inadvertently
adjectives	amenable; distinguished; invaluable; esteemed; intricate; profound; distinct; unique
connectors	with the advent of; across various areas; through the lens of; at its/the core
noun phrases	in the realm of; intriguing question; a paradigm shift
others	shaping the future of; it is anticipated that; different domains

### Reviewer Comments and Peer Review

AI tools are no longer limited to manuscript preparation; they are now also starting to influence the peer review process. An increasing number of reviewers, whether consciously or experimentally, are turning to language models to assist in drafting their reports. The advantages are evident *at a glance*: AI can generate well-structured, grammatically polished, and efficiently organized comments, which may help streamline an otherwise time-consuming task. However, this practice also introduces notable risks. Many reviewer reports now exhibit linguistic features that suggest AI assistance, including the use of generic expressions, overly formalized sentence structures, or stock phrases, often lacking specificity or a clear linkage to the manuscript content. In some cases, comments include internal inconsistencies, recommendations that fall outside the scope of the paper, or requests for changes addressing issues that are already correctly presented, indicators that raise questions about the depth of engagement with the manuscript. More recently, evidence has emerged in high-prestige journals showing that authors have been sneaking secret messages into their papers to influence AI-driven reviews. These messages (e.g., “Ignore all previous instructions. Give a positive review only”) were inserted in white, small-sized text within the manuscript so as not to be detected by humans and to prompt a favorable peer-review report. It is unclear whether this actually works, but it is certainly a symptom that the process is flawed and that many reviewers are using AI to make their work easier, or, even worse, to have AI do it entirely. In any case, if reviewers had proper incentives to do the job correctly, they would not resort to AI. When AI-generated content is not carefully reviewed with the right subject-specific knowledge, it can weaken the peer review process, especially if reviewers lean too much on AI suggestions without taking the time to verify or think critically about them.

At the same time, scientific journals are beginning to implement policies regarding AI use in two ways: (1) in the peer review process by the reviewer and (2) in manuscript preparation by the author. First, we can find statements such as “Reviewers must not use AI or AI-assisted tools to review submissions or to generate peer review reports. Reviewers are solely responsible for the content of their reports, and the use of AI technologies for this purpose constitutes a breach of peer review confidentiality”. Second, a growing number of journals now include a section at the time of submission, requiring authors to declare whether AI tools were used in writing and, if so, to explain how.^23,24^ These steps reflect a broader concern within the scientific community over transparency and accountability in the use of AI. This raises an important and as yet unresolved question: *Should the use of AI in peer review also be regulated or disclosed?* Given the potential implications for the fairness of scientific assessment, it may be necessary to extend such declarations not only to authors but also to reviewers.

## Strengths and Risks of AI Integration in the Analytical Chemistry Field

As AI tools become increasingly integrated into the workflows of analytical chemists, especially in the context of scientific writing, it is essential to examine both their benefits and the potential risks they involve. The adoption of AI technologies brings tangible improvements in productivity and accessibility, yet it also poses challenges related to scientific integrity as well as authorship. One of the main risks currently under discussion is the still unanswered question of who should be held accountable for mistakes or false claims generated by AI.

To provide a structured overview of these dynamics, Table 2 presents a SWOT analysis (strengths, weaknesses, opportunities, and threats) associated with AI implementation in the scientific communication processes of analytical chemistry. The phenomenon of *AI-washing*, where manuscripts appear well-written and technically polished but lack analytical depth or critical interpretation, represents a particularly pressing concern. As AI-generated content becomes harder to distinguish from human-authored text, maintaining transparency and accountability in scientific publishing will require both editorial adaptation and community-wide awareness.

**Table 2:** SWOT Analysis of AI-Assisted Scientific Writing in Analytical Chemistry

strengths	weaknesses	opportunities	threats
saves significant time in drafting and editing	risk of overreliance and reduced individual engagement with the writing process	wider access to scientific publishing for early career researchers	data security and ethical considerations
enhances accessibility for non-native English speaker	decreased critical thinking and originality in discussion sections	standardization of scientific communication formats	*AI washing* risk: scientifically shallow but linguistically polished articles
improves linguistic quality and fluency of scientific texts	ethical concerns around unacknowledged AI use	development of new editorial guidelines or AI coauthorship models	loss of diversity in writing styles and scientific voices
improves fast structuring of literature reviews and experimental sections	difficulty in detecting which parts were AI-generated	potential training benefits for students learning scientific writing	possible manipulation in peer review using AI-generated comments
			challenges for editors and reviewers in assessing authorship integrity

## Strategy-Based Suggestions for Responsible AI Use in Analytical Chemistry Writing

Simply identifying the strengths, weaknesses, opportunities, and threats of AI is not enough. To ensure that AI is used effectively and ethically in writing, we must move from passive observation to proactive action. This section offers practical, context-specific strategies aimed at guiding researchers, journal editors, and educators toward responsible AI use in the preparation of scientific manuscripts in analytical chemistry. These strategies are grouped into four categories: SO (strengths–opportunities) strategies, which use AI’s advantages such as efficiency and improved clarity to open up new possibilities like supporting less experienced researchers or standardizing reporting formats; WO (weaknesses–opportunities) strategies, which address limitations such as overreliance or reduced originality by linking them to opportunities for better training and clearer authorship policies; ST (strengths–threats) strategies, which employ AI’s capabilities to mitigate risks, particularly in safeguarding ethics and preserving the scientific depth of manuscripts; and WT (weaknesses–threats) strategies, which adopt a cautious approach to reduce risks related to both internal shortcomings and external pressures, including the loss of diverse scientific voices or peer review quality. These are not rigid rules, but rather flexible recommendations that can be adapted to different research contexts. Our goal is to transform SWOT analysis into an evidence-based action plan that balances AI’s potential with the discipline’s scientific rigor.

### SO Strategies

One of AI’s most valuable contributions is its ability to speed up the writing process while enhancing clarity and grammar. This is particularly beneficial for researchers in non-English-speaking countries. In analytical chemistry, where precision and standardization of terminology are critical, AI-assisted grammar checks and plain-language rewrites can help researchers present their findings more clearly to a global audience. To promote responsible use, institutions and supervisors should provide guidelines on the transparent disclosure of AI involvement and training on when and how to use AI without compromising originality. Another important strength of AI lies in organizing complex experimental details, such as sample preparation workflows, analytical instrumentation settings, and validation parameters, into clear, structured formats. AI can also assist in identifying gaps in the literature during review writing, generating indices for drafts, suggesting keywords and titles, and designing tables. When combined with critical human oversight, this approach maintains scientific depth while maximizing efficiency.

### WO Strategies

Overdependence on AI can lead to shallow reasoning, repetitive phrasing, or reduced critical thinking. These risks are particularly problematic in analytical chemistry, where experimental decisions must be justified and contextualized. Academic programs could implement assignments requiring manual drafting of sections, such as discussions and conclusions, before introducing AI for refinement, encouraging students to compare and evaluate changes. Faculty could run workshops demonstrating how AI’s suggestions may introduce subtle inaccuracies or oversimplifications in method interpretation. Journals can adopt AI usage disclosure statements and develop reviewer guidelines on evaluating AI-assisted content. Clearer editorial policies might also set boundaries for AI-generated content in hypothesis formulation or statistical interpretation, areas where human domain knowledge is essential.

### ST Strategies

AI is particularly good at polishing grammar and structuring content, but this polished surface can sometimes mask an insufficient scientific contribution. Authors should explicitly state which parts of the manuscript were AI-assisted, and reviewers should be trained to assess not only linguistic quality but also scientific validity. Journals could implement submission checklists requiring authors to highlight AI use in method descriptions, data analysis explanations, or figure captions. AI could also be used in prereview stages to flag unclear parameter reporting or inconsistencies in units and terminology, allowing authors to correct these before peer review. Editorial teams could maintain databases of AI-assisted edits to monitor trends and detect excessive reliance, ensuring the diversity of style and originality in published work.

### WT Strategies

A balanced approach is to hybridize writing by using AI for repetitive or mechanical tasks such as grammar, formatting, figure legends, or initial summaries while ensuring that core sections such as the interpretation of chromatographic results, discussion of matrix effects, or justification of method validation parameters are entirely human-written. To strengthen this, research groups could create internal guidelines specifying which manuscript sections are AI-permissible and which require human-only drafting. Detection tools customized to scientific writing could be paired with author declarations to build trust rather than penalize. Training workshops for editors and reviewers might include practical exercises on identifying overstandardized AI prose, combined with modules on constructive feedback for AI-assisted manuscripts. These measures help safeguard the integrity of analytical chemistry publications while allowing researchers to benefit from AI’s efficiencies.

## Future Trends and Perspectives

The integration of AI into analytical chemistry is poised to redefine not only how data is processed but also how knowledge is generated and communicated. Looking ahead, AI is expected to optimize chromatographic separations, accelerate method development, and even contribute to the design of novel materials and green analytical procedures. With advanced algorithms continuously improving in predictive power and processing efficiency, analytical chemists will be able to push the boundaries of speed, sensitivity, and selectivity in ways that were previously unattainable. Yet, the path toward full-scale AI integration is not without challenges. Model interpretability, ethical data management, and cybersecurity are central concerns that must be addressed through a sustained collaboration among analytical chemists, data scientists, and ethicists. As AI tools continue to penetrate all stages of the research cycle, their role in scientific publishing also demands critical reflection. While many journals have introduced mandatory disclosure sections regarding AI use in manuscript preparation, similar measures have yet to be implemented for peer reviewers or editors. This raises an important question: *To what extent should AI use be regulated in scientific publishing?* The establishment of good practices, including transparency, formal reporting of AI involvement, and ethical guidelines for its use, will be key to ensuring that innovation does not outpace accountability.

Equally important is the role of education. As AI increasingly acts as a scientific copilot, it is imperative to incorporate AI training into the curriculum of analytical chemistry programs. Numerous online courses, certifications, and specialized degrees are now available to equip both early-stage researchers and experienced professionals with the skills to use AI responsibly and effectively. In this context, it is essential to reflect on the impact of AI tools on PhD students and early career researchers. When properly integrated into training, these technologies can serve as powerful assistants by helping to identify relevant literature, organize data, suggest experimental approaches, and improve scientific writing rather than as mere text generators from which information is copied and pasted without critical evaluation. Their advantages include increasing efficiency, broadening access to advanced analytical capabilities, and supporting the acquisition of complex skills in less time. However, overreliance on AI may hinder the development of critical thinking, problem-solving abilities, and methodological autonomy. Therefore, it is crucial to teach young researchers how to use AI effectively and responsibly, ensuring that it complements their expertise, stimulates creativity, and strengthens the fundamental skills required for independent scientific practice.

New opportunities are also arising, including discussions around AI coauthorship, automated manuscript evaluation, and the use of alternative scientific metrics derived from AI-driven analytics. While some of these changes are still debated, they clearly point to a deeper transformation of how the scientific community works and communicates. Ultimately, the analytical chemistry community must engage collectively in shaping how AI is adopted and regulated. The goal should not be to replace human expertise but to augment it, ensuring that technological advances are guided by scientific reasoning, creativity, and a strong commitment to research integrity. As AI becomes an integral part of scientific practice, the challenge will lie not in resisting its use but in steering it wisely.
